# MicroRNA-21 Promotes Proliferation of Fibroblast-Like Synoviocytes through Mediation of NF-*κ*B Nuclear Translocation in a Rat Model of Collagen-Induced Rheumatoid Arthritis

**DOI:** 10.1155/2016/9279078

**Published:** 2016-06-26

**Authors:** Ying Chen, Pei-Feng Xian, Lu Yang, Sheng-Xu Wang

**Affiliations:** Department of Acupuncture and Moxibustion, School of TCM, Southern Medical University, Guangzhou 510515, China

## Abstract

MicroRNA-21 (miR-21) is overexpressed in patients with rheumatoid arthritis (RA). This study was designed to investigate the effect and mechanism of miR-21 on cell proliferation in fibroblast-like synoviocytes (FLS) of RA. FLS were primary-cultured from a rat RA model. RA-FLS and normal FLS were infected with lentivirus (anti-miR-21 or pro-miR-21) for overexpression or downregulation of miR-21, respectively. The effects of miR-21 overexpression or inhibition on nucleoprotein NF-*κ*B levels and FLS cell proliferation were evaluated by western blotting and MTT assays. The effects of an inhibitor of NF-*κ*B nuclear translocation (BAY 11-7082) were also evaluated. The results showed that the levels of miR-21 and nucleoprotein NF-*κ*B were increased in FLS of RA model rats compared to the control group. Downregulation of miR-21 in RA FLS led to a significant decrease in nucleoprotein NF-*κ*B levels and cell proliferation rates compared to the antinegative control (NC) group. However, miR-21 overexpression in normal FLS resulted in a significant increase of nucleoprotein NF-*κ*B levels and cell proliferation rates compared to the pro-NC group. The effects of miR-21 overexpression were reversed by BAY 11-7082. We concluded that upregulated miR-21 in FLS in RA model rats may promote cell proliferation by facilitating NF-*κ*B nuclear translocation, thus affecting the NF-*κ*B pathway.

## 1. Introduction

Rheumatoid arthritis (RA) is a chronic progressive inflammatory disease characterized by inflammation in the joint lining and destruction of cartilage and bone [1]. RA significantly impacts the quality of life and can lead to functional impairment and disability, affecting not only individual patients but also their family and society [2]. Fibroblast-like synoviocytes (FLS), which can be permanently altered and can orchestrate destruction of the cartilage as well as participating in synovial inflammation, are the primary effectors of cartilage destruction in RA [3]. The contribution of FLS to RA in the synovial intimal lining is especially interesting, and its mechanisms of action are still uncertain. Therefore, understanding the molecular mechanisms underlying the progression of RA, especially in FLS, remains a major unmet need.

Recently, studies of microRNAs (miRNAs) have opened new opportunities in disease research [4, 5]. miRNAs are a class of small noncoding RNAs that regulate a wide variety of gene expression, promoting mRNA degradation or inhibiting translation [6]. Increasing numbers of reports suggest that aberrant miRNA expression is connected with various diseases and cancers, regulating many biological processes such as cell proliferation [7]. Additionally, studies have revealed that aberrant miRNA expression is involved in the pathogenesis of RA [8, 9]. miR-21, the most commonly upregulated miRNA in various cancers, is upregulated in blood and T cells of patients with RA [10, 11]. Dong et al. demonstrated that miR-21 might contribute to the imbalance of Th17 and Treg cells [9]. Conversely, nuclear factor-kappaB (NF-*κ*B) is a family of ubiquitously expressed transcription factors (TFs) that play a significant role in most immune and inflammatory responses [12]. Okazaki et al. reported that an inhibitor of inhibitory *κ*B proteins (I*κ*B) could suppress the nuclear translocation of NF-*κ*B in FLS and this compound could inhibit the proliferation of rheumatoid FLS and be effective against collagen-induced arthritis in mice [13]. Moreover, Li et al. found that inhibition of miR-21 could significantly decrease NF-*κ*B activity and that overexpression of miR-21 could reverse NF-*κ*B activity inhibition and apoptosis induced by resveratrol in glioma cells [14]. However, there are few studies of the linkage between NF-*κ*B and miR-21 in RA FLS. Thus, it would be interesting to explore whether miR-21 expression could affect NF-*κ*B activity in FLS and to elucidate the underlying mechanisms involved in RA pathogenesis.

Collagen-induced arthritis (CIA) is a well-established experimental model of human RA [15]. CIA can be established by injecting type II collagen (CII) together with adjuvant in rats, leading to a disease that resembles RA [16]. In this study, FLS were primary-cultured from RA model rats. miR-21 and the nucleoprotein NF-*κ*B levels in RA-FLS were evaluated. Moreover, we explored the effects of downregulation or overexpression of miR-21 on nucleoprotein NF-*κ*B levels and FLS viability. We sought to investigate whether miR-21 had any effects on NF-*κ*B activity and FLS proliferation rate in RA and to gain more insights into the mechanisms of RA progression.

## 2. Materials and Methods

### 2.1. Animals and RA Model Preparation

Twelve male Wistar rats (obtained from the local Animal Center) weighing 160–180 g were used in this study. The rats were maintained under specific pathogen-free (SPF) conditions, fed standard chow and allowed tap water ad libitum. The animals were randomized into 2 groups (the RA group and the normal control) with 6 rats in each group after feeding adaptively for 7 days. All experimental protocols adhered to guidelines of Animal Care and were approved by the local institutional review boards.

CIA model induction with an injection of collagen emulsion was conducted as previously reported [17]. Briefly, native bovine CII was dissolved in 0.01 M acetic acid at a concentration of 4 mg/mL with gentle stirring at 4°C overnight. The solution was then emulsified with the same volume of complete Freund's adjuvant. The rats in the experimental group were immunized by intradermal injection at the base of the tail and the back with 0.25 mL of collagen emulsion. The mice were boosted 7 days later by the same method. The hind paw thickness was measured and the signs of arthritis were observed. Clinical signs such as hind paw swelling and redness were assessed according to a scoring system [18].

### 2.2. Isolation and Culture of FLS

After the successful construction of the RA model, synovial tissues were obtained and were cut into blocks of 1 mm × 1 mm × 1 mm in Dulbecco's modified Eagle's medium (DMEM) under sterile conditions. The tissues were aspirated using a sterile pipette and sprayed evenly onto the bottom of the 25 cm^2^ flasks. Then, 4 mL DMEM supplemented with 10% fetal calf serum (FCS) was added to the flask and the cultures were kept at 37°C in 5% CO_2_. The medium was replaced after 1 day. The tissue blocks were washed with PBS one week later and after the primary culture reached 70% confluence, the cells were passaged. After three passages, the cultures consisted of 98% FLS (a homogeneous population), as confirmed by microscopic analysis.

### 2.3. Lentivirus Preparation and Transduction in Cells

We investigated the effect of manipulating miR-21 levels in cells using lentiviruses encoding pro-miR-21, anti-miR-21, or control (empty plasmid). The packaged lentiviruses and corresponding empty vectors were purchased from Jikai Biotechnical Company (Shanghai, China). The packaged lentiviruses were named pro-miR-21 and anti-miR-21. The corresponding negative control (NC) was named pro-NC or anti-NC and theoretically had no effect on any gene. For transduction of recombinant lentiviral particles, the third-passage cells at the logarithmic growth phase were suspended and seeded into 12-well plates at a density of 1 × 10^6^ cells/well. The cells were then transduced with anti-miR-21 or pro-miR-21 at a multiplicity of infection (MOI) of 20 and then were allowed to recover in complete fresh medium for an additional 24 h. Because the lentiviral vectors express green fluorescent protein (GFP), the infection efficiency was assessed by GFP fluorescence microscopy at 72 h after infection. Quantitative real-time PCR (Q-PCR) analysis was performed to determine the miR-21 expression level in the transduced cells.

### 2.4. Quantification of miR-21 Expression

miR-21 expression was quantified using the Q-PCR System (Foster City, California, United States) to verify regulation of the miR-21 targets. The target miR-21 expression was normalized to U6. Total RNA from cells in the 6-well plates was isolated using TRIzol reagent (Invitrogen, USA) and reverse-transcribed to cDNA using the PrimeScript® miRNA cDNA synthesis kit (Perfect Real Time. TaKaRa, Japan) in accordance with the manufacturers' instructions. PCR was then used to amplify miR-21 using SYBR Premix Ex TaqTM II (Perfect Real Time, TaKaRa, Japan) and miR-21-specific primers (forward, 5′-TAGCTTATCAGACTGATGTTGA-3′ and reverse, 5′-AGTGCGTGTCGTGG-3′, RiboBio, Guangzhou, China) at 95°C for 5 min, followed by 40 cycles of 95°C for 15 seconds and 60°C for 60 seconds. The primers used to amplify U6 were 5′-GCTTCGGCAGCACATATACTAAAT-3′ (forward) and 5′-CGCTTCACGAATTTGCGTGTCAT-3′ (reverse). All reactions were carried out in triplicate and were repeated 3 times. Cycle threshold (Ct) values were used to analyze expression data with the 2^−ΔΔCt^ method [19].

### 2.5. Western Blotting Analysis

The cells were treated with pro-miR-21, anti-miR-21, anti-NC, and/or inhibitor of NF-*κ*B (BAY 11-7082, purchased from Calbiochem, USA) and were lysed to extract the nucleoproteins. Briefly, the nucleoproteins were extracted from cells grown in 60 mm sterile dishes in accordance with the manufacturer's instructions (KGP150, Nanjing KeyGen Biotech, Nanjing, China). Protein concentrations were determined using the Bradford method. Equal amounts of protein were then separated by 10% sodium dodecyl sulfate-polyacrylamide gel electrophoresis (SDS-PAGE) for 40 min at 110 V together with a size marker. The proteins were subsequently transferred to a polyvinylidene fluoride (PVDF) membrane (Bio-Rad, Hercules, CA) in standard transfer buffer containing glycine, methanol, Tris-base, and SDS. After transfer, the PVDF membrane was exposed to a blocking buffer of 5% bovine serum albumin (BSA) at 37°C for 2 h. The membranes were incubated overnight at 4°C with the primary antibodies of rabbit anti-NF-*κ*B and mouse anti-histone (1 : 1000 dilution, both from Cell Signaling Technology, Beverly, MA, USA). The membrane was washed 3 times with TBST for 5 min. Subsequently, the blots were incubated with horseradish peroxidase- (HRP-) labelled goat anti-rabbit or anti-mouse secondary antibody (1 : 15000 dilution, Bio-Rad) for 1.5 h at room temperature. The membrane was then washed with TBST 4 times for 15 min once. The bands were visualized by enhanced chemiluminescence according to the instructions (Millipore) and analyzed using Image Lab version 2.0.1 (Bio-Rad). The scanned images were semiquantified via Quantity One software (Bio-Rad Laboratories, Milan, Italy). The experiment was carried out 3 times.

### 2.6. Cell Viability Assay

Cell viability was determined using the MTT assay (MTT: 3-[4,5-dimethyl-thiazolyl-2]-2,5-diphenol tetrazolium biomide) (Sigma). In brief, cells in logarithmic growth phase were collected and suspended and then seeded into 96-well plates at a density of 1 × 10^3^–1 × 10^4^ cells/well (100 *μ*L/well). Various concentrations of drug were added on the second day. Five wells were run for each concentration. The cells were cultured at 37°C in 5% CO_2_ for 12–48 h and then 20 *μ*L of 5 mg/mL MTT reagent (Sigma) was added into each well for 1 h at 37°C. One hour later, 150 *μ*L dimethyl sulfoxide (DMSO) was added into every well. The plates were then placed on a shaking table for 10 min to solubilize crystals adequately. The absorbance at 490 nm was determined using an automatic enzyme-linked immunosorbent assay reader (ELx800, BioTek Instruments, USA). The zero wells (culture medium without cells, MTT, DMSO) and the control wells (cells, same concentration of drug solution, cell culture medium, MTT, DMSO) were set. The spectrophotometer was calibrated to zero absorbance using the zero wells and the relative cell viability related to the control wells was calculated. The 50% inhibition concentration (IC_50_) was calculated according to the improved Karber method [20] using the following formula: lgIC_50_ = *X*_*m*_ − *I*[*P* − (3 − *P*_*m*_ − *P*_*n*_)/4], where *X*_*m*_: lg (maximum dose), *I*: lg (maximum dose/adjacent dose), *P* is sum of the positive response rates, *P*_*m*_ is maximum positive response rate, and *P*_*n*_ is minimum positive response rate. The experiment was repeated 3 times.

### 2.7. Statistical Analysis

All of the statistical analyses were undertaken using SPSS software, version 16.0 (SPSS, Chicago, IL). The data are expressed as mean ± standard deviation (SD). Differences were analyzed using one-way analysis of variance (ANOVA) [21]. The homogeneity of the variance was tested using the Levene test [22], followed by post hoc tests using Fisher's protected least significant difference test (LSD) [22]. *P* < 0.05 was considered to be a statistically significant difference.

## 3. Results

### 3.1. Determination of Nucleoprotein NF-*κ*B and miR-21 Levels in FLS of RA and Normal Control Groups

**Figure 1:**
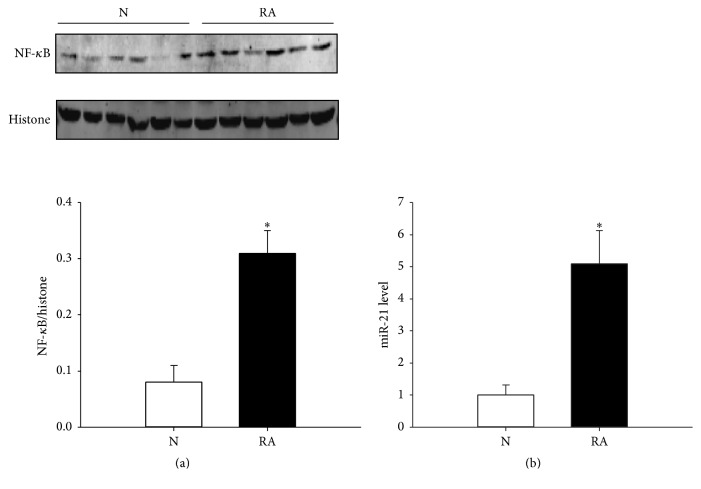
Evaluation of miR-21 and nucleoprotein NF-*κ*B levels in the RA group and normal control. (a) NF-*κ*B expression pattern in the RA group and normal control. (b) The miR-21 level was detected by Q-PCR in the RA group and normal control. N indicates the normal control. RA represents the RA group. ^*∗*^ *P* < 0.05. The results were statistically significant.

To compare the nucleoprotein NF-*κ*B and miR-21 levels between FLS in the RA and normal control groups, we examined the levels of nucleoprotein NF-*κ*B and miR-21 in primary cells using western blotting and Q-PCR methods, respectively. The results revealed that the levels of nucleoprotein NF-*κ*B in the RA group (0.31 ± 0.04) were significantly higher than those in the normal group (0.08 ± 0.03) (*P* < 0.05), as shown in Figure 1(a). Similarly, the results of Q-PCR showed that the levels of miR-21 in the RA group (5.09 ± 1.04) were significantly higher than in the normal group (1.00 ± 0.32) (*P* < 0.05) (Figure 1(b)).

### 3.2. Effect of miR-21 Inhibition on Nucleoprotein NF-*κ*B Level and FLS Proliferation Rate

**Figure 2:**
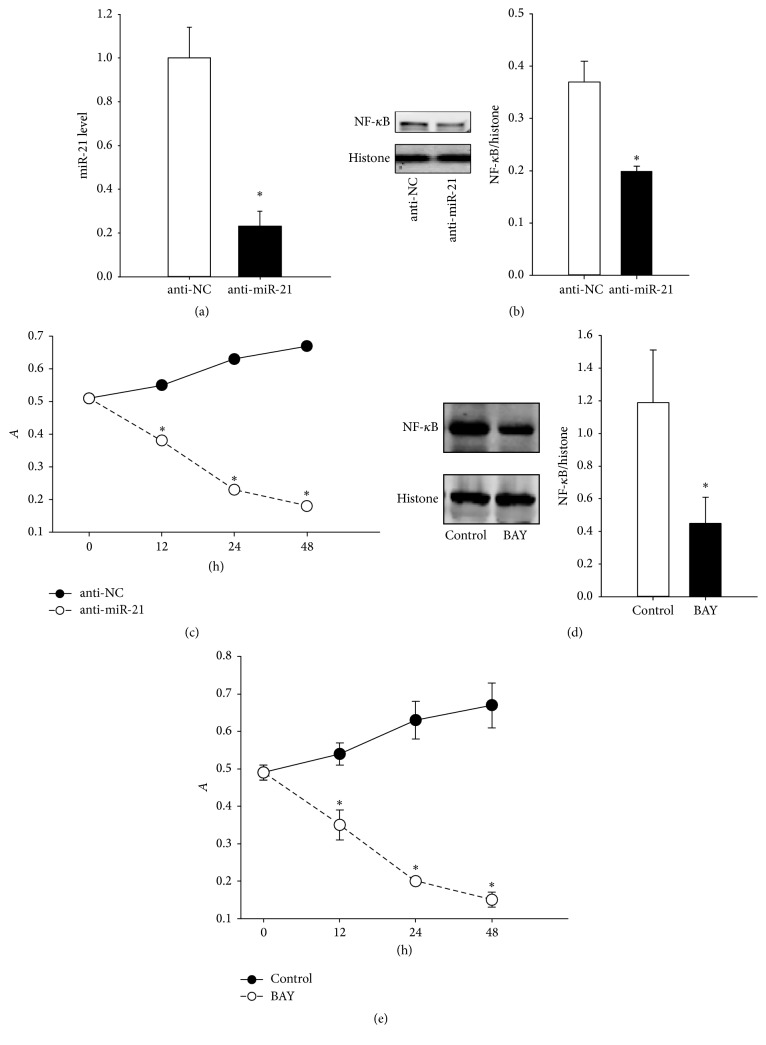
The effects of downregulated miR-21 on nucleoprotein NF-*κ*B level and cell viability. (a) The miR-21 levels in RA FLS after anti-miR-21 treatment. (b) The determination of nucleoprotein NF-*κ*B levels using western blotting methods. (c) The change in the RA FLS proliferation rate after anti-miR-21 treatment. (d) The nucleoprotein NF-*κ*B level after BAY 11-7082 treatment in RA FLS. (e) The change in the FLS proliferation rate after BAY 11-7082 treatment in RA FLS. ^*∗*^ *P* < 0.05. The results were statistically significant.

To explore the effects of the lower miR-21 level, the RA-FLS were infected with anti-miR-21 or anti-NC packaged lentiviruses. Compared with the anti-NC group, the inhibition of miR-21 expression led to a reduction in miR-21 and nucleoprotein NF-*κ*B levels as determined by western blotting analysis and Q-PCR assay, respectively (Figures 2(a) and 2(b)). Specifically, as shown in Figure 2(a), the miR-21 level was reduced in cells transfected with anti-miR-21 (0.23 ± 0.07) compared to anti-NC (1.00 ± 0.14) (*P* < 0.05). In addition, the reduction in miR-21 levels by treatment with anti-miR-21 reduced the level of nucleoprotein NF-*κ*B (0.20 ± 0.01) compared to the anti-NC group (0.37 ± 0.04) (*P* < 0.05). The FLS proliferation rate was determined by the MTT assay (Figure 2(c)). A significant difference was observed between the cells with anti-miR-21 treatment at 12 h (0.38 ± 0.04), 24 h (0.23 ± 0.01), or 48 h (0.18 ± 0.02) and cells treated with anti-NC at 12 h (0.55 ± 0.03), 24 h (0.63 ± 0.05), or 48 h (0.67 ± 0.06) (*P* < 0.05).

To elucidate the involvement of NF-*κ*B mediated by miR-21, we pretreated the FLS with BAY 11-7082, a compound known to inhibit NF-*κ*B nuclear translocation. As indicated in Figure 2(d), in the presence of BAY 11-7082, the levels of the nucleoprotein NF-*κ*B (0.45 ± 0.16) were significantly inhibited compared to those in the anti-NC group (1.19 ± 0.32) (*P* < 0.05). In addition, as shown in Figure 2(e), MTT analysis revealed a significant difference between the cells treated with BAY 11-7082 at 12 h (0.35 ± 0.04), 24 h (0.20 ± 0.01), or 48 h (0.15 ± 0.02) and cells treated with anti-NC at 12 h (0.54 ± 0.03), 24 h (0.63 ± 0.05), or 48 h (0.67 ± 0.06) (*P* < 0.05).

### 3.3. Effect of miR-21 Overexpression on Nucleoprotein NF-*κ*B Levels and FLS Proliferation Rate

**Figure 3:**
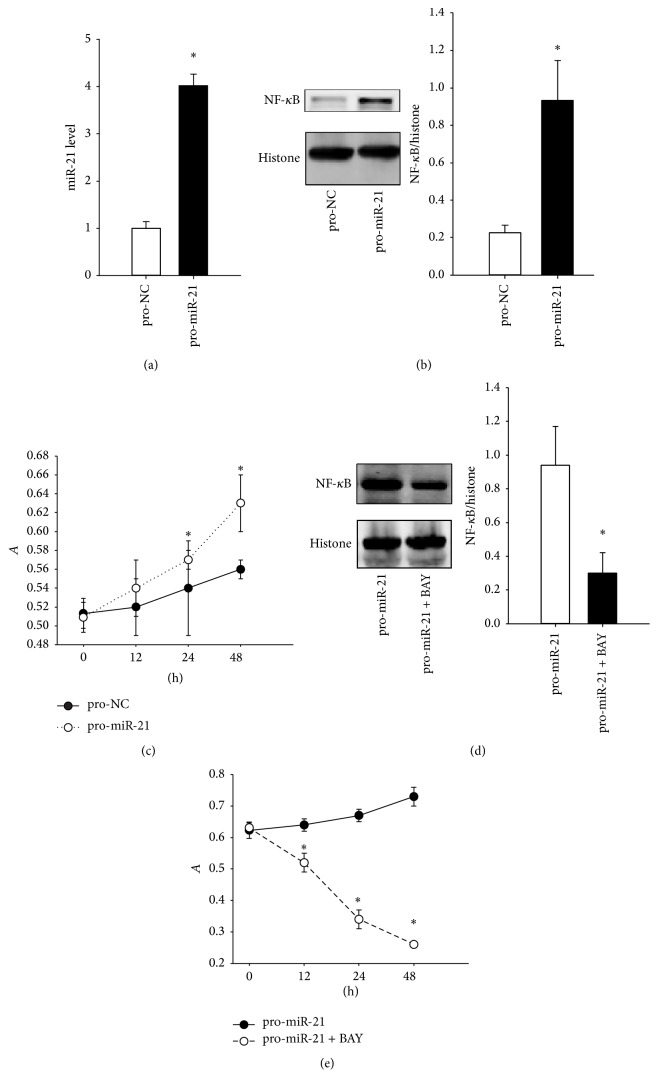
The effects of overexpressed miR-21 on nucleoprotein NF-*κ*B level and cell viability. (a) The miR-21 levels in normal FLS after pro-miR-21 treatment. (b) The determination of nucleoprotein NF-*κ*B levels using western blot methods. (c) The change of normal FLS proliferation rate after pro-miR-21 treatment. (d) the nucleoprotein NF-*κ*B level detection after additional BAY 11-7082 treatment in normal FLS. (e) The change in FLS proliferation rate after the additional BAY 11-7082 treatment in normal FLS. ^*∗*^ *P* < 0.05. The results were statistically significant.

To measure the effect of the increased miR-21 level, the normal FLS were infected with pro-miR-21 or pro-NC packaged lentiviruses. As shown in Figure 3(a), the miR-21 level as determined by Q-PCR was upregulated in cells transfected with pro-miR-21 (4.02 ± 0.25) compared to cells transfected with pro-NC (1.00 ± 0.14) (*P* < 0.05). Additionally, as shown in Figure 3(b), increased miR-21 levels by treatment with pro-miR-21 elevated the nucleoprotein NF-*κ*B level (0.94 ± 0.21) compared to the pro-NC group (0.23 ± 0.03), as measured by western blotting (*P* < 0.05). The cell viability was determined by MTT assay (Figure 3(c)). The cell viability in the group with pro-miR-21 treatment at 12 h (0.54 ± 0.03), 24 h (0.57 ± 0.01), or 48 h (0.63 ± 0.03) was different from the pro-NC group at 12 h (0.52 ± 0.03), 24 h (0.54 ± 0.05), or 48 h (0.56 ± 0.01). However, only a significant difference was found at 48 h (*P* < 0.05).

Furthermore, in the presence of BAY 11-7082, the levels of nucleoprotein NF-*κ*B in the pro-miR-21+BAY 11-7082 group (0.30 ± 0.12) were significantly inhibited compared to the group treated only with pro-miR-21 (0.94 ± 0.23) (*P* < 0.05) (Figure 3(d)). In addition, as shown in Figure 3(e), the results of MTT analysis revealed a significant reduction in the cells treated with pro-miR-21+BAY 11-7082 at 12 h (0.52 ± 0.03), 24 h (0.34 ± 0.03), or 48 h (0.26 ± 0.01) compared to cells treated with pro-miR-21 at 12 h (0.64 ± 0.02), 24 h (0.67 ± 0.02), or 48 h (0.73 ± 0.03) (*P* < 0.05).

## 4. Discussion

To better understand the molecular mechanism of RA progression, the dysregulation of miRNAs is an intriguing area [8]. Recently, miR-21, which is upregulated in a variety of diseases, was reported to be upregulated in blood and T cells of RA patients [10, 11]. In this study, we found that miR-21 and the nucleoprotein NF-*κ*B were upregulated in FLS of RA model rats. In addition, the overexpression of miR-21 induced an increase in the nucleoprotein NF-*κ*B levels and FLS proliferation rate, while miR-21 inhibition resulted in decreased nucleoprotein NF-*κ*B levels and reduced cell viability of FLS.

miR-21 has been reported to play a large role in the development of some human diseases and cancers, such as lupus [23] and lung fibrosis [24]. With regard to tissue remodeling, Thum et al. reported that miR-21 could stimulate MAP kinase signaling in fibroblasts and cause cardiac fibroblast survival and cardiac remodeling [25]. Previous studies have suggested that miR-21 could play an essential role in modulating cell proliferation [26, 27]. In this study, we found that the expression levels of miR-21 in FLS from RA model rats were higher than normal FLS using Q-PCR detection. In addition, the proliferation of RA-FLS was significantly inhibited when the cells were infected with lentivirus encoding anti-miR-21 to inhibit the miR-21 levels. Moreover, the FLS proliferation rate was obviously facilitated when normal FLS were treated with pro-miR-21 to cause miR-21 overexpression. Hence, our data suggested that altered expression of miR-21 may play a crucial role in FLS proliferation in RA progression.

The transcription factor NF-*κ*B plays a crucial role in inflammation and cell proliferation and survival [28]. Roman-Blas and Jimenez reported that NF-*κ*B was abundant in the rheumatoid synovium and that NF-*κ*B inhibition might be a rational target in the treatment of RA [12]. In our study, the levels of nucleoprotein NF-*κ*B in RA-FLS were significantly higher than in normal FLS. In addition, inhibition of NF-*κ*B nuclear translocation led to the inhibition of FLS proliferation, in accordance with the findings of Alghasham and Rasheed [29]. Because NF-*κ*B and miR-21 levels were significantly upregulated in RA-FLS, we considered the possibility that NF-*κ*B and miR-21 were connected in some way, which was supported by the study of Shin et al. [30]. They reported that miR-21 might be directly regulated by the transcription factor NF-*κ*B. In accordance with this idea, we carried out the miR-21 overexpression and inhibition experiments and investigated the effect on nucleoprotein NF-*κ*B levels and FLS proliferation rate. Interestingly, our results showed that overexpression of miR-21 resulted in higher nucleoprotein NF-*κ*B levels. In this regard, overexpression of miR-21 may promote NF-*κ*B nuclear translocation. Similarly, inhibition of miR-21 expression levels significantly decreased NF-*κ*B nuclear translocation, suggesting that miR-21 may have a stimulating effect on NF-*κ*B nuclear translocation. Furthermore, after treatment with the NF-*κ*B nuclear translocation inhibitor BAY 11-7082, the pro-miR-21-induced NF-*κ*B nuclear translocation was significantly inhibited; FLS proliferation was also inhibited. Taken together, we suggested that miR-21 might promote FLS proliferation via facilitating NF-*κ*B nuclear translocation.

In conclusion, our study demonstrated that miR-21 might promote NF-*κ*B nuclear translocation and NF-*κ*B signaling pathway activation, thus accelerating FLS proliferation, which plays a central role in RA progression. Our findings provide more insight into the molecular mechanisms of RA progression. However, further investigations are needed to clearly define this concern and facilitate our current understanding, which would be helpful for the development of novel and effective treatment strategies.

## Additional Points

The highlights of this study are as follows:

1. miR-21 and nucleoprotein NF-*κ*B levels were upregulated in FLS in a rat model of RA.
2. Inhibition of miR-21 levels in RA-FLS reduced the nucleoprotein NF-*κ*B level and FLS proliferation rate.
3. miR-21 overexpression in normal FLS led to increased nucleoprotein NF-*κ*B levels and cell viability.
4. The effects of miR-21 overexpression were reversed by BAY 11-7082.
